# Study on the Discrimination of Possible Error Sources That Might Affect the Quality of Volatile Organic Compounds Signature in Dairy Cattle Using an Electronic Nose

**DOI:** 10.3390/vetsci9090461

**Published:** 2022-08-29

**Authors:** Asmaa S. Ali, Joana G. P. Jacinto, Wolf Mϋnchemyer, Andreas Walte, Björn Kuhla, Arcangelo Gentile, Mohamed S. Abdu, Mervat M. Kamel, Abdelrauf Morsy Ghallab

**Affiliations:** 1 Department of Theriogenology, Faculty of Veterinary Medicine, Cairo University, Giza P.O. Box 12211, Egypt; 2 Department of Veterinary Medical Sciences, University of Bologna, Ozzano dell’Emilia, 40064 Bologna, Italy; 3 AIRSENSE Analytics GmbH, 19061 Schwerin, Germany; 4 Research Institute for Farm Animal Biology (FBN), Institute of Nutritional Physiology ‘Oskar Kellner’, 18196 Dummerstorf, Germany; 5 Department of Animal Management and Behavior, Faculty of Veterinary Medicine, Cairo University, Giza P.O. Box 12211, Egypt

**Keywords:** electronic nose, exhaled breath, non-invasive analysis, precision livestock farming, precision medicine, VOCs

## Abstract

**Simple Summary:**

In recent decades, remarkable progress in the development of electronic nose (EN) technologies, particularly for disease detection, has been accomplished through the disclosure of novel methods and associated devices, mainly for the detection of volatile organic compounds (VOCs). Herein, we assessed the ability of a novel EN technology (MENT-EGAS prototype) to respond to direct sampling and to evaluate the influence of possible error sources that might affect the quality of VOC signatures. Principal Component Analyses (PCA) evidenced the presence in the analyzed samples of sufficient information to consent the discrimination of different environmental backgrounds, feed headspaces and exhalated breath between two groups of cows fed with two different types of feed. Moreover, discrimination was also observed within the same group between exhalated breaths sampled before and after feed intake. Based on these findings, we provided evidence that the MENT-EGAS prototype can identify error sources with accuracy. Livestock precision farming technologies are powerful tools for monitoring animal health and welfare parameters in a continuous and automated way.

**Abstract:**

Electronic nose devices (EN) have been developed for detecting volatile organic compounds (VOCs). This study aimed to assess the ability of the MENT-EGAS prototype-based EN to respond to direct sampling and to evaluate the influence of possible error sources that might affect the quality of VOC signatures. This study was performed on a dairy farm using 11 (*n* = 11) multiparous Holstein-Friesian cows. The cows were divided into two groups housed in two different barns: group I included six lactating cows fed with a lactating diet (LD), and group II included 5 non-lactating late pregnant cows fed with a far-off diet (FD). Each group was offered 250 g of their respective diet; 10 min later, exhalated breath was collected for VOC determination. After this sampling, 4 cows from each group were offered 250 g of pellet concentrates. Ten minutes later, the exhalated breath was collected once more. VOCs were also measured directly from the feed’s headspace, as well as from the environmental backgrounds of each. Principal component analyses (PCA) were performed and revealed clear discrimination between the two different environmental backgrounds, the two different feed headspaces, the exhalated breath of groups I and II cows, and the exhalated breath within the same group of cows before and after the feed intake. Based on these findings, we concluded that the MENT-EGAS prototype can recognize several error sources with accuracy, providing a novel EN technology that could be used in the future in precision livestock farming.

## 1. Introduction

There is an increasing world-wide awareness that artificial intelligence (AI) in livestock may play an important role in the management [1]. Since 2000 BC, both the Greeks and the Chinese have used the olfactory system to diagnose diseases [2]. More recently, AI devices (colloquially called electronic nose, EN) were developed with the aim of mimicking the olfactory system [3,4,5]. These devices are made up of chemical sensors combined with a pattern recognition system [6,7]. These chemical sensors produce an electrical signal (similar to nerve cells) mimicking biological olfactory receptors [8]. The obtained signals are subsequently analyzed by pattern recognition software that is able to classify and memorize odors resembling the biological cerebral cortex of the brain [3,4,5].

Contrary to conventional odor analysis methodologies in the laboratory, EN has been developed for applications that demand rapid and precise measurements. EN technologies are promising tools in a large spectrum of fields such as robotics [9,10,11,12], environment monitoring [13,14,15,16], food engineering [17,18,19,20,21], disease diagnosis [22,23,24,25,26] and animal management [27,28,29].

Several studies on cattle exhaled breaths, such as volatile organic compounds (VOCs) using an EN, have been carried out [7,8,30,31]. In cattle, EN has been applied in metabolomics for methane production evaluation [32], detection of estrus [27,28,29,31], disease diagnosis (e.g., bovine respiratory disease, ketosis) [8,30] and identification of potential biomarkers [33,34]. Most of these studies aimed either to test the EN capability for taking representative and reproducible exhalated breath samples with minimal stress to the animal and/or discriminating between diseased and healthy cattle [31,32]. Few studies have investigated the influence of error sources that might affect exhalated breath sampling (e.g., source sampling distance, air turbulence, head movement, and eructation). However, systematic measurement errors might affect the accuracy of the results [35].

Ali & Ali (2020) developed a prototype based on EN technology, the so-called Milking Machine and Electronic Nose Technology- Egypt, and the Asmaa Shaaban prototype (MENT-EGAS) [36,37]. MENT-EGAS is based on 10 non-specified chemical metal-oxide sensors. By using the pattern generated from these sensors and various algorithms, the device can identify up to 10 different compounds or to provide a straightforward answer, such as “Good” or “Bad” and “Yes” or “No,” depending on the needs of the user. This prototype was originally developed for cattle estrus detection based on perineal odor. Therefore, this study aimed to assess the ability of the MENT-EGAS prototype to respond to direct sampling and to evaluate the influence of possible error sources that might affect the quality of VOC signatures.

## 2. Materials and Methods

### 2.1. Instrumentation and Sampling Measurements

The MENT-EGAS prototype (Patent No. WO2010099800A2) provided by AIRSENSE ANALYTICS GmbH (Schwerin, Germany) was used to measure VOCs. In this study, a “Yes” or “No” approach was applied.

**Figure 1:**
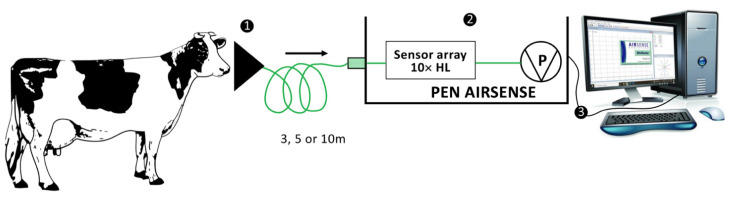
Schematic diagram for MENT-EGAS prototype setup for exhaled breath sample collection and its three main units: (**1**) collecting unit, (**2**) detection, analysis and identification unit, and (**3**) results analyzing unit.

It consisted of three main units: (**1**) the collecting unit; (**2**) the detecting, analyzing and identification unit; and (**3**) the results analyzing unit (Figure 1).

**Figure 2:**
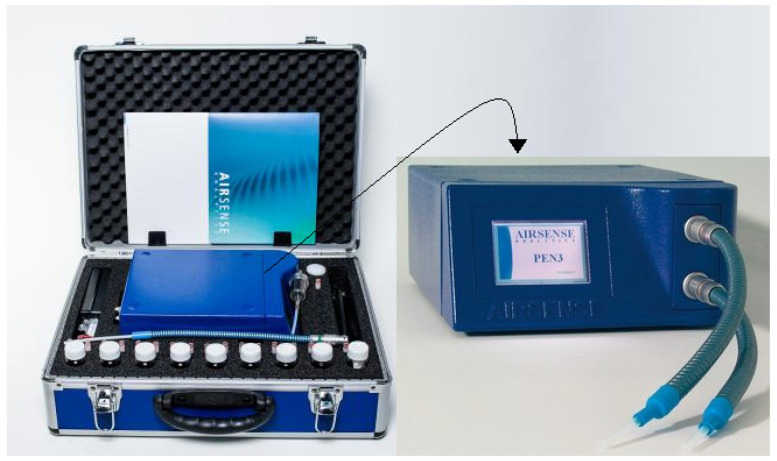
Portable electric nose version 3.5 (PEN 3.5).

The collecting unit was represented by a funnel connected through a 2 m long Teflon tube to the second unit. The detecting, analyzing and identification unit was represented by a portable EN with responses of 10 metal-oxide sensors version 3.5 (PEN 3.5) (Figure 2). The results analyzing unit was represented by the database Winmuster Software, Version 1.6.2.22 Copyright© AIRSENSE ANALYTICS GmbH.

All collected samples were measured by the same operator and device to minimize variations and to control extra factors that might cause measurement errors.

For each sample, three consecutive measurements were conducted. Each measurement had a duration of 40 s and a cleaning phase of 60 s. In each measurement, three vectors (36, 37 and 38 s from a total of 40 s sampling duration) were obtained and appended to establish a pattern for further analysis with principal component analysis (PCA).

### 2.2. Animals, Diet and Housing

The experiment was performed at a dairy farm at the Institute of Nutritional Physiology Oskar Kellner, Research Institute for Farm Animal Biology (FBN), Dummerstorf, Germany. Eleven (*n* = 11) multiparous (2 to 4 parities) Holstein-Friesian dairy cows ranging from 3.5 to 5.5 years old were used in this study. A preliminary clinical examination was carried out in order to exclude any respiratory, digestive or metabolic disorders as well as mastitis.

The cows were divided into two groups:

- Group I included six lactating cows (*n* = 6), who were fed twice daily (6:00 am and 4:30 pm) on a conventional lactation diet (LD) (Table 1). In this group, cows were chosen randomly from healthy lactating cows, regardless of their age or days in milk (DIM).
- Group II included five (*n* = 5) non-lactating late pregnant cows (7 to 9 months of pregnancy), which were fed twice daily (6:00 am and 4:30 pm) on a conventional far-off diet (FD) (Table 1). In this group, cows were randomly chosen from healthy non-lactating cows.

All animals were housed in a free-stall, semi-closed, well ventilated system with curtained sidewalls barn. Group I and II were housed in two separate barns with an independent different entrance. Both groups had similar management (capacity, ventilation, housing type, watering, feeding and manure cleaning up).

### 2.3. Type of VOC Determination

#### 2.3.1. Environmental Background VOC Determination

**Figure 3:**
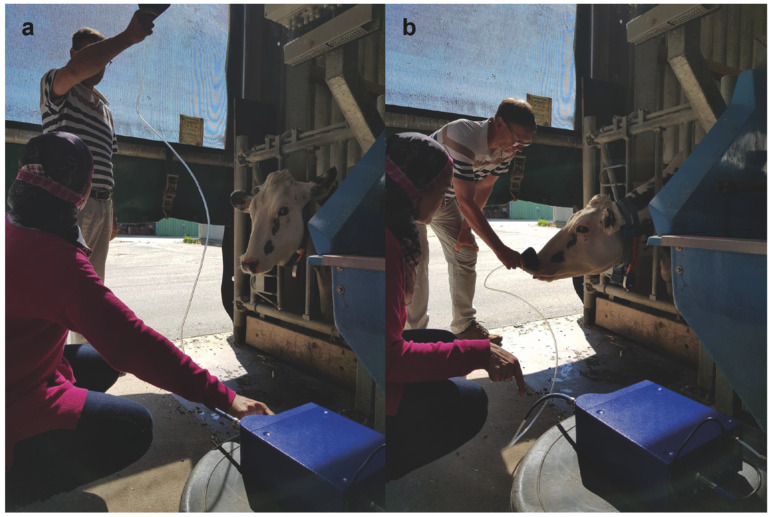
Sampling using the MENT-EGAS prototype. (**a**) Environmental sampling in the barn. (**b**) Exhaled breath sampling.

Environmental background (barn air) VOC determinations were obtained from the two barns (groups I and II) four hours after the morning meal (Figure 3a). Environmental samples were analyzed to determine the effect of the VOCs globally emanated from exhaled breath, feed headspace, manure and other possible sources of emanated gases.

#### 2.3.2. Feed Headspace VOC Determination

Feed headspace VOC determinations were obtained directly from a bucket containing the different feeds offered to the cows: LD diet, FD diet, and pellet concentrates. Feeds’ samples were analyzed to determine the effect of the VOCs emanated from the feedstuffs only.

#### 2.3.3. Exhaled Breath VOC Determination

Exhaled breath VOC determinations were carried out four hours after the morning meal.

Each group was offered 250 g of their respective diet; 10 min later, exhalated breath was collected for VOC determination. After this sampling, 4 cows from each group were offered 250 g of pellet concentrates. Ten minutes later, the exhalated breath was collected once more.

The samples were obtained by positioning the funnel in front of the cow’s muzzle (Figure 3b). Exhaled breath samples were analyzed to determine the effect of the VOCs emanating from the exhaled breath only.

### 2.4. Response to the Sensor and Data Analysis

The sensor response from 10 metal-oxide (PEN 3.5) was recorded for each sample. The measurements data were obtained from three vectors (36, 37 and 38 s from a total of 40 s sampling duration) and were analyzed with PCA.

PCA [38] was used as a preliminary comparison of VOCs emanating from LD, FD and pellet concentrates. The PCA technique was applied to reduce the dimensionality of complex obtained datasets (data from a ten-dimensional room due to the ten used sensors) into fewer dimensions, maximizing the difference between the obtained data, increasing the interpretability but at the same time minimizing information loss. Data transformation was performed, and graphical plots were obtained [39].

### 2.5. Ethics Statement

This study did not require official or institutional ethical approval, as no invasive techniques were used. All animals in this study were inspected with the consent of their owners and handled according to good ethical standards.

## 3. Results

### 3.1. Environmental Background

**Figure 4:**
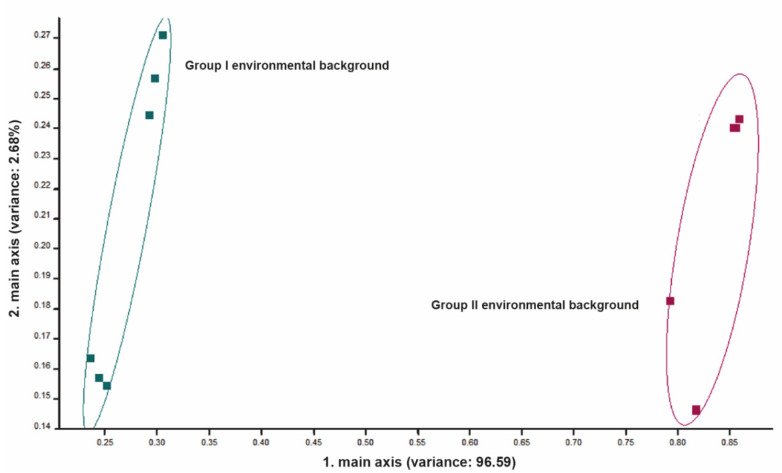
Principal component analysis (PCA) for environmental background of group I and group II. Note the high discrimination between the two environmental backgrounds. The numbers in parentheses indicate the percentages of the data matrix described by the relevant components and functions.

By measuring the surrounding environment to detect the effect of the VOCs emanated from exhaled breath, feed headspace, manure and other possible sources of emanated gases, and by applying the PCA, a clear discrimination between both environments was noticed (Figure 4). Different signals were received by the sensors in both environments (barns from Group I and II).

### 3.2. Feed Headspace

**Figure 5:**
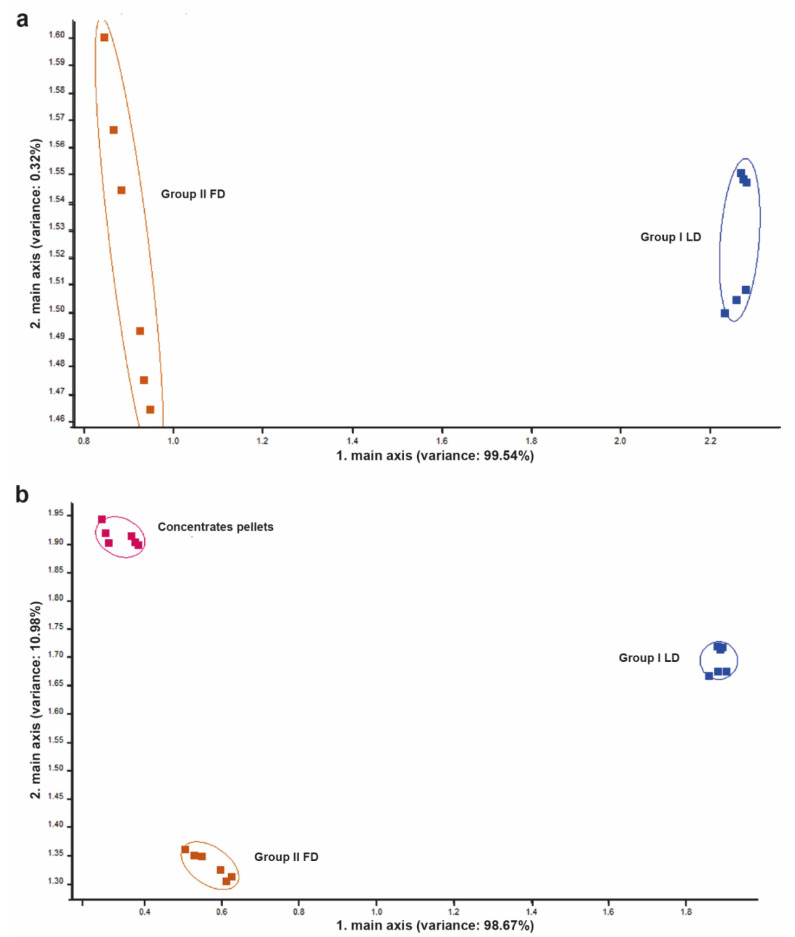
PCA for feed headspace. (**a**) PCA for lactational diet (LD) in group I and for far-off diet (FD) in group II. Note the high discrimination between the two types of feed. (**b**) PCA for LD in group I, for FD in group II and pellet concentrates. Note the high discrimination between the three samples. The numbers in parentheses indicate the percentages of the data matrix described by the relevant components and functions.

By measuring the feed headspace to detect the effect of the VOCs emanating from it and by applying the PCA, a clear discrimination between headspace samples from LD and FD diets was noticed (Figure 5a). Moreover, when comparing the pellet concentrate headspace with both the LD and FD headspace samples, the PCA showed high discrimination between LD and FD. In addition, a high discrimination between LD and FD was observed when compared to the pellet concentrates (Figure 5b).

### 3.3. Exhaled Breath

**Figure 6:**
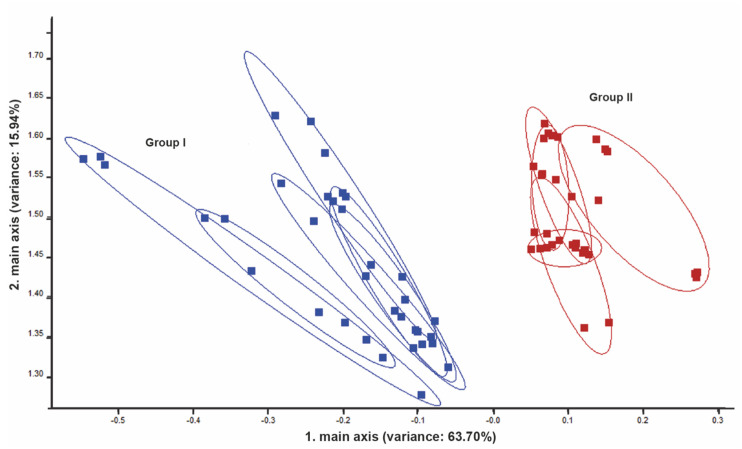
PCA for exhalated breath for cows from groups I and II after 250 g of LD and FD ingestion, respectively. Note the high discrimination between the two groups. The numbers in parentheses indicate the percentages of the data matrix described by the relevant components and functions.

PCA of exhalated breath revealed discrimination between group I and II after 250 g LD and FD ingestion, respectively. The PEN 3.5 system was able to discriminate between the two groups without overlapping when considering individual variations (Figure 6).

**Figure 7:**
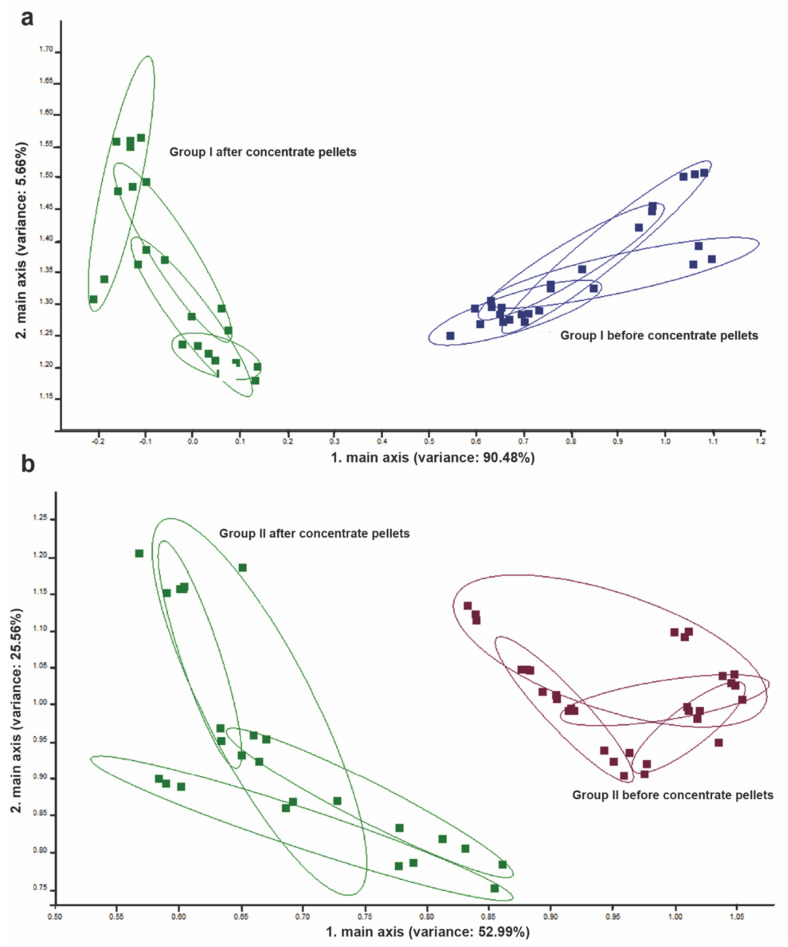
PCA for exhalated breath of cows before and after ingestion of pellet concentrates. (**a**) PCA for exhalated breath of cows from group I before and after the ingestion of pellet concentrates. (**b**) PCA for exhalated breaths of cows from group II before and after the ingestion of pellet concentrates. The numbers in parentheses indicate the percentages of the data matrix described by the relevant components and functions.

Considering only group I, PCA of breath revealed discrimination between exhaled VOCs measured after 250 g of LD and after 250 g of LD and pellet concentrate ingestion (Figure 7a). Furthermore, among group II, similar to group I, PCA of breath revealed discrimination between exhaled VOCs measured after 250 g of FD and after 250 g of LD and pellet concentrate ingestion (Figure 7b).

## 4. Discussion

In this study, the EN-based MENT-EGAS prototype was able to respond to direct sampling. The PCA analyses demonstrated that there was adequate information present in the samples to consent to discrimination between (1) two different environmental backgrounds, (2) different feed headspaces, (3) exhalated breath from two groups of cows with a different type of feed, and (4) exhalated breath from the same group of cows before and after ingestion of pellet concentrates. These findings demonstrate that the MENT-EGAS prototype is able to differentiate with accuracy different types of samples.

In recent decades, several studies on EN technologies in the agriculture and veterinary fields have been performed [3,7,8,30,31,32]. However, the application of EN technology in livestock precision agriculture and veterinary medicine still has some limitations related mostly to the influence of possible error sources that might affect the quality of VOC signatures [31,40,41]. Different factors might affect the accuracy of the EN measurements, such as farm-to-farm variation, cow-to-cow variation, diet-to-diet variation, productive phase of the animal (in lactation or dry), breed of the animals and day-to-day variation [42]. These error factors could be avoided if accurately managed before applying EN technologies, where the correct recognition of error factors is fundamental to obtain reliable results. Most previous studies reporting the use of EN technologies in the agriculture and veterinary fields have been affected by some of these problems [7].

The environmental background, as well as the feed headspace, when evaluating exhalated breath in cattle are very important error factors that should be taken into consideration. The animal’s exhalated breath after exhalation was diluted with barn air at the sampling point, representing an error source when analyzing the samples [43,44]. In addition, the VOC composition of rumen gas and the effects of burping on the VOC composition of respiratory air should be considered when analyzing the respiratory air from cattle [44,45,46,47].

Even though cow-to-cow variation has been considered an error factor in previous studies [10,30,31], in our study, we could not recognize this variation within cows with the same feed and within the same phase of production (Group I vs. Group II).

Metabolic physiologic processes, depending on feed and the phase of production, can alter the composition of exhalated VOCs [48,49,50,51]. Some studies suggest that among growing, lactating, and non-lactating cattle and between dairy and beef cattle fed the same feed, there are no significant differences in methane emissions [52,53,54]. On the contrary, methane emissions differ for cows in different phases of production and between dairy and beef cattle when the diet composition varies [48,50,51]. In the particular case of high-producing cows, there is an increase in methane emissions because they have a high dry matter intake and are fed with digestible low fiber diets compared with non-lactating cows [48,50,51,52]. This was the case in this study, where we observed a clear discrimination between VOCs from different feed headspaces and in exhalated breath from cows of group I (lactating cows on an LD) and group II (non-lactating cows on an FD), as well as between exhalated breath from cows of the same group before and after ingestion of pellet concentrates.

## 5. Conclusions

In this study, we demonstrate that the MENT-EGAS prototype is able to recognize with accuracy several error sources such as the environmental backgrounds, feed headspaces and exhaled breath from cows with different types of diet. Therefore, we provide evidence that this novel non-invasive EN technology could be used in the future as a valid tool in precision agriculture and in precision livestock farming. In particular, MENT-EGAS might be used in the future for disease diagnosis, such as metabolic or respiratory disorders or for estrus detection. However, further studies need to be performed.

## Figures and Tables

**Table 1:** Detailed lactation diet (LD) and far-off diet (FD) composition offered to cows of group I and group II, respectively.

Lactation Diet (LD)		Far-Off Diet (FD)	
Feed Constitute	Mass (kg, Organic Matter)	Feed Constitute	Mass (kg, Organic Matter)
Gras silage	5.00	Gras silage	5.00
Gras silage	7.00	Gras silage	7.00
Corn silage	26.00	Corn silage	15.00
Barley straw	1.00	Barley straw	1.50
Concentrate	6.00	Concentrate	3.00
Rapeseed extraction meal	1.20	Rapeseed extraction meal	0.5
Wheat	0.46	Wheat	1.00
Soybean extraction meal	0.46	Hay	1.00
Corn	1.64	Minerals	0.22
Minerals	0.16		
Lime	0.09		

## Data Availability

The data that support the findings of this study are available from the corresponding author upon request.
